# Evaluation of miRNA-16–2-3P, miRNA-618 levels and their diagnostic and prognostic value in the regulation of immune response during SARS Cov-2 infection

**DOI:** 10.1007/s00251-023-01308-6

**Published:** 2023-05-24

**Authors:** Nourelhoda E. Hassan, Walaa A. Moselhy, Ehab B. Eldomany, Emad Farah Mohamad Kholef

**Affiliations:** 1 Laboratory Department, Luxor Fever and GIT Hospital, Luxor, Egypt; 2 Department of Biotechnology and Life Sciences, Faculty of Postgraduate Studies for Advanced Sciences, Beni-Suef University, Beni-Suef, Egypt; 3 Department of Clinical Pathology, Faculty of Medicine, Aswan University, Aswan, Egypt

**Keywords:** COVID-19, Interleukin-1β (IL-1β), Interleukin-8 (IL-8), miRNA-16–2-3P, miRNA-618

## Abstract

Following the announcement of the pandemic of COVID-19 in December 2019, several studies focused on how to early predict the severity of the disease in symptomatic and asymptomatic patients. Many cytokines including interleukin-6, interleukin-8, and tumor necrotic factors have been concluded as strong indicators for COVID-19 infection. Additionally, miRNAs have been associated with dysregulation in the immune system. The aim of this study are the following: (1) to estimate the level of miRNA-16–2-3P, miRNA-618, IL-8, IL-1β as predictors for SARS-CoV-2 complications in PCR negative and positive patients; (2) to assess the biological role and effect of these miRNAs on SARS-CoV-2 pathogenicity. Our study showed that the level of IL-1β had been significantly associated with patient who need hospitalization, also the alteration of the level of miRNA-16–2-3P, miRNA-618 is positively correlated with the admission of these patients and influence the outcomes of SARS-cov-2 infection. Measurement of miRNA-16–2-3P, miRNA-618, IL-1β could be a good predictor of COVID-19 patient outcome. However the measurement of IL-8 levels during immune responses in the admitted and in ICU patients could have a prognostic value.

## Introduction

As of February 2021, the COVID-19 pandemic, caused by infection with severe acute respiratory syndrome-associated coronavirus-2 (SARS-CoV-2), has resulted in over 107 million cases and 2.35 million deaths worldwide (Farr et al. 2021). In Egypt, starting from the 3rd of January 2020 to the 21st of February 2022, 469,457 recorded cases of COVID-19 with 23,752 deaths have been detected (WHO 2022). The outcome of SARS-CoV-2 infection varies widely from asymptomatic to severe disease associated with acute respiratory distress syndrome (ARDS) and death (Wu et al. 2020). Several studies have established that host responses to infection play a critical role in determining disease outcomes in infected patients.

A significant decrease in lymphocytes were lower than 20% in severe cases has been detected (Del Valle et al. 2020), specifically, CD4 + T, CD8 + T, natural killer (NK) cells, and B cell number (Fara et al. 2020; Huang et al. 2020).

Many reviews have evaluated the associated inflammatory mediators with COVID-19. For example, hyper-inflammatory responses including high levels of circulating cytokines and chemokines (particularly interleukin (IL)-6, IL-8, and tumor necrosis factor (TNF)-α) (Xu et al. 2020), IL-10 and C-reactive protein (Del Valle et al. 2020), IL-17 (Darif et al. 2021), and IL-18 (Valizadeh et al. 2020); lymphopenia and immune cell infiltration in infected organs are considered major determinants of COVID-19 severity (Xu et al. 2020). Interleukin-8 (IL-8) is an effective pro-inflammatory cytokine that has been associated with the enrollment and activation of neutrophils during inflammation. Therefore, detected neutrophilia detected in COVID-19 patients can contribute to the pathophysiology of the disease (Feldmann et al. 2020).

The characterization of host factors associated with SARS-CoV-2 pathogenesis is critically important for the design of novel therapies.

MicroRNAs (miRNAs) are a class of non-coding RNAs that regulate endogenous gene expression at the post-transcriptional level. In most instances, miRNAs function by interacting with the 3′ untranslated region (3′ UTR) of target mRNAs to induce degradation and translational repression (Bartel 2018). There are currently over 2600 human miRNAs listed in the miRNA registry (miRBase, version 22), which are estimated to collectively regulate 60% of all human protein-coding genes (Farr et al. 2021).

Some studies have revealed that the viral miRNAs can modify some of the host inflammatory responses to constrain secure damage to susceptible organs including—lungs (Panda et al. 2022). On the other hand, the host miRNAs have been proved to inhibit viral replication via self-attachment at 3′UTR region of the viral genome or the cellular targeting receptor or blocking the structural and non-structural proteins of SARS-CoV-2 without disturbing the expression of the human genome (Chow and Salmena 2020). Therefore, miRNAs could modulate the immune response of the severely infected COVID-19 patients via different mechanisms.

Li et al. (2020) data analysis suggests that, 35 miRNAs were upregulated and 38 miRNAs were downregulated in the human patients with COVID‐19. The top genes were as follows: miR‐16‐2‐3p, miR‐6501‐5p, and miR‐618 were highly expressed in COVID‐19 patients and that miR‐183‐5p, miR‐627‐5p, and miR‐144‐3p were less expressed in COVID‐19 patients.

The scientific rationale for investigating miRNAs during viral infections is two-fold. Firstly, miRNA profiles offer unique insight into cellular pathways associated with virus replication and pathogenesis. For instance, the human coronavirus OC43 potentiates NF-kB activation during infection by binding and sequestering miR-9, a negative regulator of NF-kB (Lai et al. 2014). There is also evidence that coronaviruses co-opt the host miRNAs response to subvert antiviral immune responses. Infection by the, *Alpha Corona Virus* transmissible gastroenteritis virus (TGEV), downregulates miR-30a-5p expression, which disrupts the type I interferon response against TGEV (Ma et al. 2018).

Secondly, the characterization of host miRNAs responses to virus infection informs the development of biomarkers for improved disease detection and forecasting of disease outcome (Tribolet et al. 2020). Several pathogenic viruses, including SARS-CoV-1, induce changes to the circulating host miRNA profile (Tambyah et al. 2013).

Viral infection can regulate miRNA expression and that can cause other genes to regulate the host immune response to viral infection. MicroRNAs effect is on virus replication and they have recently emerged as important modulators of viral infections (Canatan and Sanctis 2020).

While it is recognized that the host response to infection plays a critical role in determining the severity and outcome of COVID-19, the host microRNA (miRNA) response to SARS-CoV-2 infection is poorly defined. The host response to SARS-CoV-2 infection provides insights into both viral pathogenesis and patient management (Farr et al. 2021).

## Patient methods and subject study

### Patients

This study was carried out in Luxor Fevers Hospital. It includes eighty patients aged from 18 to 70 years suffering from typical COVID-19 symptoms. Patients under the study have been divided into two groups according to the RT-PCR results:

- Group 1: seventy cases with proved positivity for COVID-19 disease Sixty are not in need for ICU admission
  - ◦ Ten of these cases are ICU admitted, and
  - ◦ Sixty are not in need for ICU admission

- Group 2: with proved negativity for COVID-19 disease using RT-PCR

Patient written informed consents were obtained from participated patients according to Helsinki Declaration. Also, approval from the Ministry of Health, Training and Research Sector has been obtained.

### Methods

1. Nasopharyngeal swab samples have been done for all patients under study for testing of COVID-19 by RT-PCR.
2. Laboratory investigations

Blood samples collected: EDTA sample, Citrate samples and samples without anticoagulants (for collection of serum samples)

All groups under study have been subjected for the following:

- Complete blood count (CBC) has been done on automated cell analyzer DXH 520 (Beckman Coulter).
- Biochemical tests (including renal function tests (urea and creatinine) and liver function tests (bilirubin (T and D), proteins (total and albumin), AST, ALT, ALP, and GGt) have been done on automated chemistry analyzer AU480 (Beckman Coulter) and electrolytes (Na and K) on ion selective electrode analyzer.
- Laboratory investigation to assess the severity of COVID-19
  - ◦ Ferritin, by chemiluminescent assay cobas e411 (Roche)
  - ◦ CRP, turbidmetric assay on automated chemistry analyzer AU480 (Beckman Coulter)
  - ◦ D-Dimer, by chemiluminescent enzyme immunoassay for the quantitative measurement of D-Dimer concentration on PATHFAST instrument
- Inflammatory cytokines (IL-1B and IL-8) using the Enzyme-Linked Immunosorbent Assay (ELISA) technique. Supplied kits from the Elabscience Biotechnology Inc. USA
- Human IL-1β and IL-8 by ELISA technique:
- Sample: Serum
- Assay procedure
- 100 μL standard and sample added to each well. Incubated for 90 min at 37 ℃
- Liquid removed. Aspirated and washed 5 times
- 100 μL Biotinylated Detection Ab has been added. Incubated for 1 h at 37 ℃
- Aspirated and washed 5 times
- 100 μL HRP conjugate added. Intubated for 30 min at 37 ℃
- Aspirated and washed 5 times
- 90 μL substrate reagent added. Incubated for 15 min at 37 ℃
- 50 μL stop solution added and read the standard and sample absorbance at 450 nm
- Plotting the standard curve
- Calculation of the results using standard curve
- MicroRNAs (miRNA-16–2-3p and miRNA-618) were assessed using Real Time-PCR.

### miRNA assay

#### RNA extraction and reverse transcription

- Total RNA, containing miRNA, was extracted from plasma samples (200 µl plasma) using the Qiagen miRNeasy Mini Kit (Qiagen GmbH, Valencia, California, USA, catalog no. 217004). The RNA purity was confirmed by the relative absorbance at 260/280 nm. Then, the extracted RNA was stored at − 80 °C and prepared for usage. Ten microliter of the extracted RNA was used for cDNA synthesis using the miScript II reverse transcription (RT) Kits (Qiagen GmbH, Valencia, California, USA), under the reactive condition of 37 °C for 60 min, then 95 °C for 5 min. Then, the miScript SYBER Green PCR kits were used with the miScript Primer Assays according to the real-time PCR Amplifier manufacturer’s instructions (Qiagen GmbH, Valencia, California, USA, catalog no. 218073). The mature miRNA-16–2-3P and miRNA-618 sequences were (CCAAUAUUACUGUGCUGCUUUA) and (AAACUCUACUUGUCCUUCUGAGU) respectively with Housekeeping gene: miRNAU6 (RNU6) as an endogenous control. For qRT-PCR, 2 μL of diluted reverse transcription products was mixed with 12.5 μL SYBR®Green Real-time PCR Master Mix. Then we add 2.5 μL of forward primer, 2.5 μL reverse primers, and 5 μL RNase-free water in a final volume of 25 μL in each strip tube. The reactivity conditions: initial activation step at 95 °C for 15 min, followed by 3-step cycling: 1-denaturation 94 °C for 15 s, 2-annealing 55 °C for 30 s, 3-extension 70 °C for 34 s. The three steps cycle has been repeated for 40 cycles.. After reaction, the threshold cycle of fluorescence (Ct) was calculated to further analyze the expression level of miRNA in specimens utilizing endogenous control. A housekeeping gene was added to each sample as an internal negative control (Catalog no. 00033712). The relative expression was expressed by 2-ΔCT (ΔCT = CT target gene-CT reference gene). The relative quantification of each of miRNA-16–2-3P and miRNA-618 was performed using quantitative real-time (qRT)-PCR according to the manufacturer’s instructions. The ΔCt value was used to represent the relative level of expression of a single miRNA.

#### Statistical analysis

Data were verified, coded by the researcher, and analyzed using the IBM-SPSS 24.0 (IBM-SPSS Inc., Chicago, IL, USA). Descriptive statistics: means, standard deviations, median, interquartile range (IQR), frequency, and percentages were calculated. Test of significances: Fisher’s Exact test was calculated to compare the frequencies among groups. The Shapiro–Wilk test will be used to test for data normality. The Student *t*-test and Mann–Whitney *U* test were calculated to test the mean/median differences in continuous variables between groups (parametric and non-parametric). Multivariable logistic regression analysis was calculated to investigate the significant predictors of positive PCR (odds ratio (OR), 95% confidence interval − 95% CI, and likelihood ratio test (LRT)). The ROC curve was depicted to explore the diagnostic performance of the new biomarkers for prediction of ICU admission, analyzed as area under the curve (AUC), standard error (SE), and 95% CI. Validity statistics (sensitivity, specificity, positive, and negative predictive value (PPV and NPV)) were calculated. The Spearman rank correlation co-efficient was calculated for univariate correlations. A significant *P* value was considered when it is equal or less than 0.05 (References: * IBM_SPSS. Statistical Package for Social Science. Ver.24. Standard version. Copyright © SPSS Inc., 2012–2016. NY, USA. 2016).

## Results

The demographic data of the two studied groups has been demonstrated in Table (1). There was no significant difference between the positive PCR COVID-19 group and negative group as regards the presence of the following diseases: asthma, chronic obstructive airway disease (COPD), hypertension (HTN), diabetes mellitus (DM), chronic kidney disease (CKD), ischemic heart disease (IHD), rheumatic heart disease (RHD), or cancer (Table 1).

**Table 1:** Baseline characteristics of the studied COVID-19 cases

	**PCR negative** **(*n* = 10)**	**PCR positive** **(*n* = 70)**	***P*-value**
**Demographic data**			
** Age/years**	48.80 ± 6.5	53.24 ± 12.3	0.499*
** Sex (male/female)**	7/3	35/36	0.131**
**Comorbidity**			
** Asthma**	5 (50%)	40 (56.3%)	0.481**
** COPD**	4 (40%)	16 (22.5%)	0.143**
** DM**	4 (40%)	27 (38%)	0.581**
**HTN**	1 (10%)	16 (22.5%)	0.249**
** IHD**	0 (0%)	4 (5.6%)	0.584**
**RHD**	0 (0%)	1 (1.4%)	0.877**
**CKD**	0 (0%)	1 (1.4%)	0.877**
**Cancer**	0 (0%)	4 (5.6%)	0.584**

*Independent *t*-test was used to compare the means among groups

**Fisher’s Exact test was used to compare the frequency among groups

Laboratory investigations of both groups revealed a significant difference regarding ALT, CRP, miRNA-618, and miRNA-16–2-3P in favor to positive PCR for COVID-19 group, as presented in Table 2.

**Table 2:** Laboratory findings of cases and control

	**PCR negative** **(*n* = 10)**	**PCR positive** **(*n* = 71)**	***P*-value**
**Laboratory findings**			
**HGB (mean ± SD)**	11.84 ± 1.6	11.53 ± 1.8	0.656*
** WBCs (median (IQR))**	7.8 (5)	6.9 (3)	0.288**
** Lymph. (median (IQR))**	1.1 (2)	0.9 (1)	0.672**
** Granular (median (IQR))**	6.3 (4)	5.5 (3.5)	0.460**
** MID (median (IQR))**	0.5 (0.4)	0.5 (0.6)	0.526**
** CRP (median (IQR))**	24 (48)	36 (36)	0.044**
** ALT (median (IQR))**	23.5 (13)	27 (17)	0.048**
** AST (median (IQR))**	21.5 (14)	26 (24)	0.127**
** D-Dimer (median (IQR))**	0.55 (0.6)	0.74 (0.4)	0.180**
** IL-1β**	165 (130)	135.5 (118)	0.061**
** IL-8**	1.81 (2)	6.2 (4)	0.080**
** miRNA-618**	0.14 (1)	1 (0.4)	0.039**
** miRNA-16–2-3P**	0.29 (1)	2.7 (5)	0.045**
** S. iron**	11 (9)	8 (3)	0.336**
** S. lead**	6.7 (2)	8 (3)	0.363**

All bold values are less than 0.05, and all bold ones are significant

*ALT* alanine aminotransferase, *AST* aspartate aminotransferase, *CRP* C-reactive protein, *S*., serum, *WBCs* white blood cell counts, *HGB* hemoglobin, *Granular* granulocytes, *Lymph.* lymphocytes, *MID* monocytes, eosinophils, basophils, blasts

*Independent *t*-test was used to compare the means among groups

**Mann Whitney *U* test was used to compare the medians among groups

A multivariable logistic regression model revealed that odd for detecting the COVID-19 infection by PCR increased to almost 3 times if the patient have COPD. Cyanosis showed 3.5 times increase in incidence for predicting the infection (*P* < 0.029), while presence of myalgia showed almost 4 times increase incidence for predicting COVID-19 infection positive PCR compared with the negative PCR cases (Table 3). Also, the incidence for prediction of COVID-19 infection increase 4 times with the level of miRNA-618 and 9 times with miRNA-16–2-3p. This may suggests that the presence of these signs or symptoms may be associated with increase viral load in the nasopharynex.

**Table 3:** Independent predictors of positive PCR among COVID-19 patients: multivariable logistic regression model

**Variable**	**Multivariate**	
	**OR (95% CI)**	***P*-value**
** Age/years**	1.014 (0.979 – 1.051)	0.442
** Sex (male)**	2.501 (0.592 – 8.522)	0.213
**COPD**	2.292 (1.002 – 9.129)	**0.044**
**Cyanosis**	3.509 (1.008 – 7.342)	**0.029**
**Epigastric pain**	0.090 (0.004 – 0.238)	**0.047**
**Myalgia**	3.664 (1.024 – 8.625)	**0.045**
**MID**	2.030 (1.080 – 8.585)	**0.036**
** miRNA-618**	4.315 (1.047 – 9.018)	**0.039**
** miRNA-16–2-3P**	9.267 (1.178 – 18.819)	**0.043**
** S. iron**	0.381 (0.111 – 0.851)	**0.027**

All bold values are less than 0.05, and all bold ones are significant

*OR* odds ratio, *CI* confidence interval

Regarding laboratory biomarkers and their association with ICU admission, ICU admitted cases show a significantly high IL-1β level in comparison to patients not in need for ICU admission. However, IL-8 level shows the reverse with a significantly high level in patients not in need for ICU admission in comparison to ICU admitted patients. Similarly, miRNA-618 level shows significant increase level in non ICU admitted patients in comparison to ICU admitted patients, while there was a marginal significant increase in the level of miRNA-16–2-3P in non ICU admitted patients. No significant difference was detected with serum iron or lead (Table 4). These biomarkers could have a prognostic value in COVID-19 infected patients.

**Table 4:** Novel markers for COVID-19 outcome

**Median (IQR)**	**ICU admission**		***P*-value**
	**No**	**Yes**	
**IL-1 β**	17.5 (14)	83 (57)	0.032*
**IL-8**	60.5 (74)	4 (14)	0.002*
**miRNA-618**	22 (43)	6 (15)	0.042*
**miRNA-16–2-3P**	7 (8)	3 (5)	0.051*
**S. iron**	8 (5)	8 (4)	0.682*
**S. lead**	9 (6)	8 (3)	0.136*

All bold values are less than 0.05, and all bold ones are significant

**Mann Whitney *U* test was used to compare the medians among groups

**Fig. 1:**
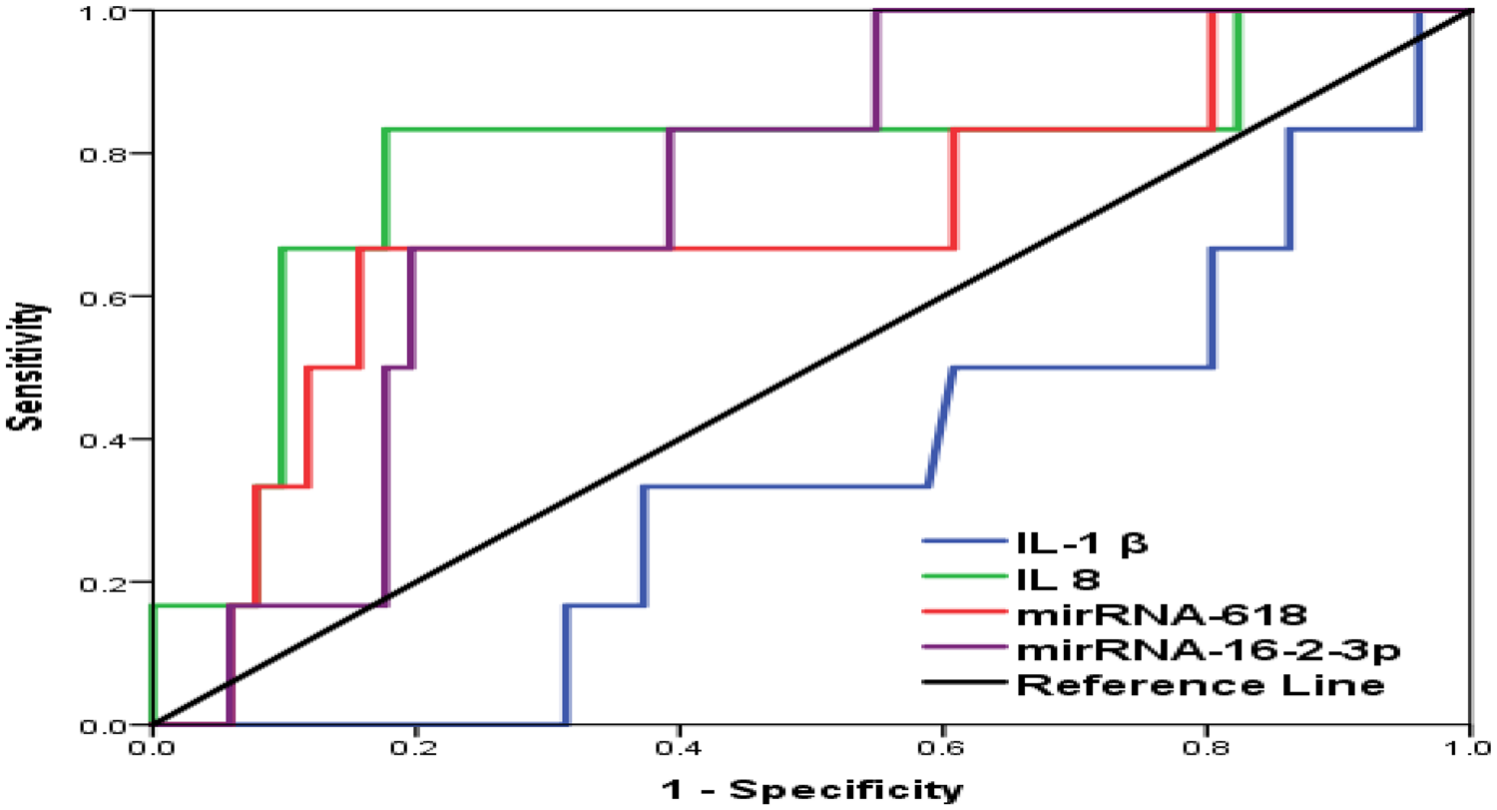
ROC curve for Biomarkers for ICU Admission Prediction

Finally, the AUC for ROC curves was used for early prediction of the severity of COVID-19. The AUC for ROC curves showed bad predictability of IL-1β (0.518 ± 0.092, CI 0.261–0.623; *P* < 0.556) for the COVID-19 infection. The cut-off values for predicting the COVID-19 was 12 pg/ml with an accuracy of 50.5%, sensitivity of 80%, and a specificity of 21% (PPV%, 50%; NPV%, 51%). However, IL-8 showed a good predictability with AUC of 0.655 ± 0.077 (CI 0.583–0.884, *P* < 0.044). The cut-off value for IL-8 was 17 pg/ml with an accuracy of 74.5%, sensitivity of 70%, and specificity of 79% (PPV%, 77%; NPV%, 72.5%). miRNA-618 AUC was 0.625 ± 0.109 (CI 0.412–0.838, *P* < 0.254). The cut-off value was 1 with an accuracy reaches 60% (sensitivity of 75% and specificity of 44%) PPV% equals 57% and NPV% of 64%. The AUC for miRNA-16–2-3P was 0.743 ± 0.078 (CI 0.591–0.895, *P* < 0.041). The cut-off values for miRNA-16–2-3P were 3.5 with an accuracy of 72.5%, sensitivity of 84%, and specificity of 61%. PPV% was 68% and NPV% of 79% (Table 5) (Fig. 1).

**Table 5:** Diagnostic criteria of biomarkers for ICU admission prediction

**Diagnostic criteria**	**IL-1 β**	**IL-8**	**miRNA-618**	**miRNA-16–2-3P**
**AUC**	**0.518**	**0.655**	**0.625**	**0.743**
**95% CI**	**0.261–0.623**	**0.583–0.884**	**0.412–0.838**	**0.591–0.895**
SE**	**0.092**	**0.077**	**0.109**	**0.078**
*P*-value***	**0.556**	**0.044**	**0.254**	**0.041**
**Cut-off**	**12 pg/ml**	**17 pg/ml**	**1**	**3.5**
**Accuracy**	**50.5%**	**74.5%**	**60%**	**72.5%**
**Sensitivity%**	**80%**	**70%**	**75%**	**84%**
**Specificity%**	**21%**	**79%**	**44%**	**61%**
**PPV%**	**50%**	**77%**	**57%**	**68%**
**NPV%**	**51%**	**72.5%**	**64%**	**79%**

*AUC area under the curve. **SE standard error, *CI* confidence interval, ***Null hypothesis true area = 0.5

Sensitivity (true positives/all diseased); specificity (true negatives/all non-diseased); PPV (true positives/all test positives); NPV (true negatives/all test negatives)

## Discussion

A negative COVID-19 test means the test did not detect the virus, but this does not rule out that you could have an infection. If you have symptoms you may have COVID-19, but tested before the virus was detectable, or you may have another illness.

Our study includes both symptomatic COVID-19 positive PCR and symptomatic negative PCR patients for COVID-19 and compare the tested cytokines (IL-1β and IL-8), and miRNA (miRNA-618 and miRNA-16–2-3p) in both groups.

In our study, positive PCR patients for COVID-19 showed no significant difference regarding clinical symptoms as sore throat, cough, fatigue, vomiting, nausea, and chills when compared to the negative PCR group for COVID-19. Although most of the cases suffered these symptoms, only myalgia and anosmia show a mild significant increase in the positive PCR group for COVID-19 in comparison to the negative PCR one; 28% of the positive PCR group had anosmia compared to non in the negative PCR group. Additionally, 32.5% of the positive PCR group had myalgia when compared to 10% of the negative PCR group (*P* < 0.05).

Early studied meta-analyses assessing the impact of comorbidities on the course and clinical outcome of COVID-19 found that about 31% of adult patients had comorbidities. Hypertension being the most prevalent condition (20.93%) followed by heart failure (10.5%), diabetes mellitus (10.4%), and coronary heart disease (8.5%) (Jutzeler et al. 2020). These pre-existing comorbidities were found to be linked with the severity of the COVID-19.

Comparison between different laboratory tests revealed a significant difference regarding only ALT and CRP in favor to cases group with no differences regarding comorbidities. In addition, the presence of COPD (OR: 3, *P* < 0.04), cyanosis (OR: 3.5, *P* < 0.029), epigastric pain had 9% chance of predicting the COVID-19 infection (*P* < 0.047) while myalgia increased the odd for prediction to almost 4 times compared with the negative cases.

Using the multivariable logistic regression model of analysis, it has been found that presence of the clinical symptoms and signs, COPD, cyanosis, epigastric pain, and myalgia, is a good predictor for COVID-19 (*P* < 0.05).

Farr et al. (2021) study stated that the host response to SARS-CoV-2 infection provides insights into both viral pathogenesis and patient management. Cytokine storm is an activation cascade of auto-amplifying cytokine production and leads to excessive activation of immune cells and the generation of pro-inflammatory cytokines (Tisoncik et al. 2012).

To lower mortality due to cytokine storm, we have to discover and find good predictors for this aggressive inflammatory response, even before its development. Several clinical studies report the infiltration of monocytes and macrophages into the lungs of the COVID-19 patients contribute to the production of pro-inflammatory cytokines and chemokines that result in cytokine storm leading to tissue damage, organ system dysfunction, and progression to ARDS as well as mortality (Costela-Ruiz et al. 2020; Gómez-Rial et al. 2020). It has been proved that the higher the level of cytokines the poor the prognosis (Chen et al. 2020, and Guohua et al. 2020). Narożna and Rubiś (2021) suggesting that COVID-2 inhibits the IFN receptor’s activation, which reduces the antiviral effect while allowing cytokine storm syndrome (i.e., IL-1, IL-6, IL-8) release. Additional study reveals a significant difference between the disease stages and different levels of IL-8 and IL-6. Also, a significant negative correlation was detected between IL-6 and IL-8 levels and SpO2, PaO2, which indicate respiratory failure. Furthermore, positive correlation between IL-8 and IL-6 and CRP was detected. The study has concluded that IL-6 and IL-8 can act as predictive biomarkers for the COVID-19 infection and severity (Li et al. 2021).

Evidence suggests that excessive inflammatory process activation leads to abundant interleukin-1β (IL-1β) release and subsequent aggravation of pulmonary injury and induce hypercoagulability, favoring progression to respiratory failure and widespread thrombosis eventually leading to multi-organ failure and death (Nicola et al. 2022).

Our data reveals that IL-1β level was significantly increase in the COVID-19 positive patients, and this level significantly increases in ICU admitted patients; this makes IL-1β a good predictor of the disease severity and patient’s outcome. In the same line with IL-1β the level of IL-8, miRNA-618 and miRNA-16–2-3p show a significantly increase level in the COVID-19 positive patients. However, the level of these parameters, IL-8, miRNA-618, and miRNA-16–2-3p, are significantly decrease in the COVID-19 ICU admitted patients in comparison to non ICU admitted one.

Our data demonstrates that the level of IL-8 significantly increase in the Covid-19 infected patients, but this level was found to decrease in ICU admitted patient in comparison to non ICU admitted patients, and these results against the recorded results by many authors who demonstrates a positive correlation between the level of IL-8 and the severity of the disease (Nagant et al. 2020; Li et al. 2021). Also, Del Valle et al. (2020) found an association between the level of IL-8 and decrease or worse survival. Our controversies results may be due to the included ICU admitted patients who are under treatment that may improve the condition and causes decrease in IL-8 level, we did not include any type of treatment in our study. So, further study includes the type of treatment and correlates this with IL-8 level may help in prediction and follow up response of the patients.

Our statistical data analysis reveals that IL-1β has a bad predictability for the COVID-19 infection. However IL-8 has a sensitivity of 70% with more specificity 79% than IL-1β with 77% PPV and 72.5% NPV. This makes IL-8 has a more diagnostic predictor for the COVID-19 patients, with an accuracy of 74.5% at a cut off value of 17 pg/ml.miRNAs are known to regulate numerous physiological pathways and biological processes including cell development, maturation, differentiation, and activation. miRNAs are vital for the regulation and elimination of undesired or malformed mRNA. miRNAs can act as anti-viral tool within the host cell as an entry for the diffusion of the virus (Abu-Izneid et al. 2021).

In our study, we compare the level of miRNA-618 and miRNA-16–2-3p between the two symptomatic groups, one with proved Covid-19 by PCR and the other group is negative for COVID-19 by PCR.

Our data analysis of the miRNA-618 and miRNA-16–2-3p levels shows a significant increase in its level in the positive PCR group in comparison to the negative PCR group (*P* < 0.05).

In one study, COVID-19 patients displayed 200 significant differentially expressed (SDE) miRNAs. Around 75 miRNAs were detected in asymptomatic patients compared to symptomatic patients who showed platelet aggregation and cytokine pathways. Moreover, the study has found that miRNAs were significantly correlated with inflammatory cytokines (Fernández-Pato et al. 2022).

One study has revealed that miRNA-618 highly expressed in patients with COVID-19 and it increased to 1.5 fold in symptomatic patients with COVID-19 compared to healthy volunteers (Li et al. 2020).

Statistical analysis of our laboratory data reveals that miRNA-618 and miRNA-16–2-3p level shows a significant increase in its level in the positive PCR for the Covid-19 group in comparison to negative PCR for the COVID-19 group (*P* < 0.05). In agreement with our results, Li et al. (2020) study reveals that miR-16–2-3p, miR-6501-5p, and miR-618 were more highly expressed in COVID-19 patients than in healthy controls.

Our study reveals that miRNA-618 and miRNA-16–2-3p level was low in ICU admitted patients in comparison to non-ICU admitted patients; however, this low level were statistically significant in miRNA-618 only (*P* = 0.042) not with miRNA-16–2-3p (*P* = 0.051). That means there is a down regulation of the miRNA-618 and miRNA-16–2-3p, and this downregulation increase with the severity of the disease and the need for ICU.

However, statistical analysis of miRNA-618 and miRNA-16–2-3p level shows it has a positive predictive value in positive COVID-19 patients. Moreover, miRNA-16–2-3p has a more sensitivity (84%) and more specificity (61%) at a cut off value 3.5 than miRNA-618 (sensitivity of 75% and specificity of 44%) at a cut off 1.0 in diagnostic prediction of COVID-19.

Our data analysis shows a negative correlation between lymphocytic percent and the level of IL-1β (-0.213 (= 0.028)). Also, this negative correlation has been statistically observed between lymphocytic differential count and miRNA-16–2-3p (-0.208 (= 0.049)). Also, there was a negative correlation between monocyte percent and miRNA-618 and miRNA-16–2-3p (-0.341 (= 0.003), -0.292 (= 0.009) respectively).

From our study we suggest that measurement of the IL-1β, IL-8, and host miRNA response could improve COVID-19 detection, outcome prediction and this potentially useful tool for early management of patients with severe forms.

Nevertheless, our study has several limitations. First, our negative PCR patients not tested for other viruses that could be the cause of symptoms. Also the number of symptomatic negative PCR cases included is low relative to positive PCR cases. Further studies would be needed to explore how the duration of illness influences the Interleukins or microRNAs level.
