# Correction to: Evaluation of miRNA-16–2-3P, miRNA-618 levels and their diagnostic and prognostic value in the regulation of immune response during SARS Cov-2 infection

**DOI:** 10.1007/s00251-023-01312-w

**Published:** 2023-07-04

**Authors:** Nourelhoda E. Hassan, Walaa A. Moselhy, Ehab B. Eldomany, Emad Farah Mohamad Kholef

**Affiliations:** 1 Laboratory Department, Luxor Fever and GIT Hospital, Luxor, Egypt; 2 Department of Biotechnology and Life Sciences, Faculty of Postgraduate Studies for Advanced Sciences, Beni-Suef University, Beni-Suef, Egypt; 3 Toxicology and Forensic Medicine Department, Faculty of Veterinary Medicine, Beni-Suef University, Beni-Suef, Egypt; 4 Department of Clinical Pathology, Faculty of Medicine, Aswan University, Aswan, Egypt

**Correction to: Immunogenetics** **https://doi.org/10.1007/s00251-023-01308-6**

The second affiliation, "Department of Biotechnology and Life Sciences, Faculty of Postgraduate Studies for Advanced Sciences, Beni-Suef University, Beni-Suef, Egypt" should have been added to the name of the co-author, Nourelhoda E. Hassan and the co-author Walaa A. Moselhy second affiliation, " Toxicology and Forensic Medicine Department, Faculty of Veterinary Medicine, Beni-Suef University, Beni-Suef, Egypt" has been added.

The original article has been corrected.

